# Changes in the Physical Properties and Volatile Odor Characteristics of Shiitake Mushrooms (*Lentinula edodes*) in Far Infrared Radiation Drying

**DOI:** 10.3390/foods12173213

**Published:** 2023-08-25

**Authors:** Long Xie, Yu-Si Jiang, Yu-Bin Wang, Hong-Wei Xiao, Wei Liu, Yue Ma, Xiao-Yan Zhao

**Affiliations:** 1 Beijing Vegetable Research Center (BVRC), Beijing Academy of Agricultural and Forestry Sciences, National Engineering Research Center for Vegetables, Key Laboratory of Urban Agriculture (North) of Ministry of Agriculture and Rural Areas, Beijing Key Laboratory of Vegetable Germplasms Improvement, Beijing 100097, China; xielong@nercv.org (L.X.);; 2 Institute of Agri-Food Processing and Nutrition, Beijing Academy of Agricultural and Forestry Sciences, Beijing Key Laboratory of Fruits and Vegetable Storage and Processing, Key Laboratory of Vegetable Postharvest Processing of Ministry of Agriculture and Rural Areas, Beijing 100097, China; 3 College of Engineering, China Agricultural University, 17 Qinghua Donglu, Beijing 100083, China

**Keywords:** shiitake mushrooms, far infrared radiation drying, microstructure, volatile compounds

## Abstract

The effects of far infrared radiation drying (FID) on physical properties (drying kinetics, color, shrinkage ratio, rehydration ratio, and microstructural characterization) and volatile odor characteristics (volatile odor profile distinction and volatile compounds) of shiitake mushrooms were evaluated in this study. During the FID, the drying time decreased with the increase in drying temperature, and it had a less significant effect in the lower temperature range. The increase in drying temperature led to increasing shrinkage and collapse in the microstructure, resulting in a decreased rehydration rate and highlighting the influence of microstructure characteristics on macroscopic properties. Higher drying temperatures employed in the FID process were found to be associated with a decreasing L* value and an increasing Δ*E* value. The application of principal component analysis can effectively distinguish the significant effect of FID on the volatile odor profiles of shiitake mushrooms. Compared to raw shiitake mushrooms, FID treatment has endowed samples with a greater variety of volatile compounds. After processing with FID, there have been increases in volatile components such as sulfur compounds, acids, nitrogen compounds, and aldehydes, while volatile components like alcohols, ketones, and hydrocarbons have shown decreases.

## 1. Introduction

Shiitake mushrooms (*Lentinula edodes*) are a highly cultivated and widely consumed species of edible fungus worldwide [1]. Shiitake mushrooms are abundant in various bioactive compounds and exhibit nutraceutical properties [2]. Raw shiitake mushrooms possess a significantly high moisture content ranging from 85% to 95%, which creates suitable conditions for the growth of microorganisms, the occurrence of chemical reactions, and enzymatic activity [3]. Due to the lack of a protective cuticle, raw shiitake mushrooms have a tender texture, making them susceptible to water loss and mechanical damage, leading to quality deterioration such as mushroom cap expansion, shrinkage, and spoilage [4]. Drying is the primary preservation method for shiitake mushrooms, effectively extending their shelf life while also imparting them with distinct flavors [5]. The drying process plays a crucial role in inhibiting the growth of spoilage microorganisms, slowing down enzyme activity, and decelerating moisture-mediated reactions [6].

Both shade-drying and hot-air-drying methods are commonly utilized for drying shiitake mushrooms due to their low capital investment and ease of operation [7]. However, the product is exposed to the open environment for an extended period, rendering it susceptible to microbial contamination, insect infestation, dust accumulation, and spoilage. Due to the high environmental humidity, it is difficult for samples to reach a safe moisture content of 13% on a wet basis, which poses a risk of spoilage during the storage period after shade-drying. Hot-air-drying presents disadvantages such as significant shrinkage and browning, the degradation of nutrition and flavor, and the alteration of the microstructure [8]. Freeze-drying preserves the primary structure and shape of products, leading to resultant products with high porosity and preferable rehydration properties [9]. The product with better quality characteristics is provided via a low-temperature and vacuum environment. The slow mass and heat transfer efficiency and maintenance of the triple-point temperature during the freeze-drying process result in low throughput and long drying time [10]. Simultaneously, freeze drying requires specialized and expensive equipment. Therefore, it is important to identify a suitable method to extend the shelf life, preserve the quality, and minimize nutrient loss during the preservation of shiitake mushrooms. Far infrared radiation drying utilizes the resonance between the radiation frequency and the inherent frequency of the moisture in materials to heat materials [11], and it has been widely used for drying biomaterials [12,13]. The far infrared radiation source releases energy that directly penetrates into the material, effectively heating both the surface and interior for ensuring consistent internal heating and swift dehydration. Simultaneous internal and external heating occurs in far infrared radiation drying. Under the effect of internal heating, the internal moisture continuously migrates to the drying medium, ensuring the smooth passage of moisture migration. However, the long wavelength in far infrared radiation drying limits the penetration depth of the energy, so it is commonly used for drying thin-layer materials.

The physical properties (drying kinetics, color, shrinkage, rehydration, and microstructural characterization) and the volatile odor characteristics (volatile odor profile distinction and volatile compounds) of shiitake mushrooms are crucial quality attributes that determine consumer acceptance and market value. The microstructural characteristics of shiitake mushrooms after drying are closely related to shrinkage and rehydration capacity, directly determining the appearance of the product after rehydration. The distinct odor characteristics of shiitake mushrooms are the result of the combined action of various volatile compounds, including alcohols, ketones, aldehydes, acids, and sulfur compounds. Xu et al. investigated the relationship between the pre-drying temperature and overall quality (shrinkage, rehydration, and color) of dried shiitake mushrooms [14]. The study conducted by Hu et al. assessed the influence of microstructure evolution on the rehydration performance of shiitake mushrooms following a process of instant controlled pressure drop combined with hot-air-drying [15]. The results were reported by Wang et al. for the effect of mid infrared drying before freeze drying and after freeze drying on the rehydration ratio, microstructure, and aroma compounds of shiitake mushrooms [16]. Zhang et al. conducted a comparison of the changes in both volatile and non-volatile flavor compounds in shiitake mushrooms during the processes of natural-drying, hot-air-drying, and freeze-drying [17].

This present study aimed to explore the impacts of far infrared radiation drying on the physical properties (drying kinetics, color, shrinkage ratio, rehydration ratio, and microstructural characterization) and the volatile odor characteristics (volatile odor profile distinction and volatile compounds) of shiitake mushrooms. This study aimed to provide theoretical evidence for the industrial far infrared radiation drying of shiitake mushrooms.

## 2. Materials and Methods

### 2.1. Material

Raw shiitake mushrooms were sourced from a local market (Beijing, China). The shiitake mushroom samples, after removing stipites, were approximately circular with similar sizes (average radius was 3.2 ± 0.4 cm). The thickness of shiitake mushroom pilei was 1.1 ± 0.2 cm. The average initial moisture content of the raw shiitake mushrooms was 87.31 ± 0.34% on a wet basis or 6.67~7.09 kg/kg on a dry basis.

### 2.2. Drying Methods

#### 2.2.1. Shade Drying (SD)

Raw shiitake mushrooms (500 g) were placed in a single layer on trays. Samples were placed in the shade and attention was given to flipping and weighting them every four hours during the day. The average temperature recorded was 25 ± 5 °C, while the relative humidity averaged 40 ± 10%. The SD process continued for approximately four days until the samples reached a constant final weight.

#### 2.2.2. Far Infrared Radiation Drying (FID)

Based on the current situation and previous studies [9,14] on shiitake mushroom drying, an appropriate temperature was set from 50 °C to 70 °C with a drying temperature interval of 5 °C. Before the experiment, the equipment was calibrated to meet the predetermined parameters. Shiitake mushrooms were arranged in a single layer on a tray measuring 30 cm × 20 cm. Each test group consisted of 12 shiitake mushrooms. The drying experiment was carried out using a far infrared radiation drying box produced by Technical Institute of Physics and Chemistry, Chinese Academy of Sciences. The single far infrared radiation heating panel, with a power rating of 300 W and a surface area of 28 × 24 cm^2^, was designed to emit thermal radiation. The adjustable temperature range was from 25 °C to 90 °C, with air flow rate of 1.5 m/s. The temperature sensor was positioned parallel to the surface of the samples for collecting the thermocouple signals. The collected thermocouple signal was utilized by the proportional integral derivative controller to regulate the operation of the heating panel. During the drying process, the sample tray was periodically taken out at 60 min intervals, and was swiftly weighed on a digital balance with an accuracy of ±0.01 g (SPS402, Ohaus Co., Parsippany, NJ, USA) and then was returned to the chamber. The drying process persisted until the moisture content of the sample reached 13% on a wet basis. After cooling to room temperature, the dried samples were packed into heat-sealed bags for subsequent measurement.

### 2.3. Drying Kinetics

The moisture ratio (MR) of shiitake mushrooms during the thin–layer drying experiments is determined using the following equation [18]:

(1) $$\mathrm{MR}=\frac{M_{t}-M_{e}}{M_{0}-M_{e}}$$

where *M*_0_ is the initial moisture content; *M_t_* is moisture content at *t* during drying (kg/kg on a dry basis); and *M_e_* is the equilibrium moisture content with a value of approximately 0.05 kg/kg on a dry basis, which is comparatively lower than *M_t_* and *M*_0_ in this test. Thus, Equation (1) can be simplified as follows [19]:

(2) $$\mathrm{MR}=\frac{M_{t}}{M_{0}}$$

### 2.4. Shrinkage Ratio

The shrinkage ratio (SR) of the dried shiitake mushrooms was determined according to Xiao et al. [3], with slight modifications. The volume replacement method using quartz powder as the medium evaluated the degree of shrinkage. The volumes of the samples were measured before drying (*V*_1_) and after drying (*V*_2_), and the shrinkage ratio was calculated using the following formula:

(3) $$\mathrm{SR}\;(\%)=\frac{V_{1}-V_{2}}{V_{1}}\times 100$$

### 2.5. Rehydration Ratio

The rehydration ratio (RR) of the dried shiitake mushrooms was determined using the method of Sun et al. [20], with slight modifications. A quantity of dried shiitake mushrooms was chosen and immersed in deionized water maintained at a constant temperature of 60 °C and then weighed at 10 min intervals until a stable weight was achieved. The formula used to calculate the rehydration rate was as follows:

(4) $$\mathrm{RR}=\frac{\mathrm{Weight}\;\mathrm{of}\;\mathrm{sample}\;\mathrm{after}\;\mathrm{rehyfration}\;(g)}{\mathrm{Weight}\;\mathrm{of}\;\mathrm{dry}\;\mathrm{sample}\;(g)}$$

### 2.6. Microstructure

Scanning electron microscopy (SU8010, Hitachi Co., Ltd., Tokyo, Japan) was used to observe the microstructure of shiitake mushroom pilei under various drying conditions [21]. The thin section from each dried sample was fixed onto a carbon adhesive tape and was gold-coated before analysis, and the acceleration voltage was 10.0 KV. Microstructure images of the samples were analyzed at 200× magnifications.

### 2.7. Color Measurements

The color parameters L*, a*, and b* were directly measured with a colorimeter (CM-700D, Konica Minolta, Inc., Tokyo, Japan), and the CIELAB color space diagram was utilized to represent sample colors following the previously described method [22]. The illuminant and observation angle are standard D65 and 10°, respectively. The total color difference value (Δ*E*) was calculated using Equation (5) [20].

(5) $$\Delta E=\sqrt{{\left(L^{*}-{L_{0}}^{*}\right)}^{2}+{\left(a^{*}-{a_{0}}^{*}\right)}^{2}+{\left(b^{*}-{b_{0}}^{*}\right)}^{2}}$$

The reference values for raw shiitake mushrooms were *L*_0_, *a*_0_, and *b*_0_. Additionally, L*, a*, and b* were used to quantify the lightness, the redness/greenness, and the yellowness/blueness of the dried samples, respectively.

### 2.8. Volatile Odor Profile Distinction

The volatile odor characteristics of shiitake mushroom pilei under different drying conditions were distinguished with electronic nose (PEN3, Win Muster Airsense Analytics Inc., Schwerin, Germany), which was equipped with 10 sensors (W1W, W2W, W1C, W3C, W5C, W1S, W2S, W3S, W5S, and W6S) capable of detecting different compounds [23]. Before the experiment, 1 g of shiitake mushrooms was added into a 30 mL sealed headspace vial and incubated at 30 °C for 30 min. During the experiment, a syringe needle was employed to puncture the sealed vial and extract volatile gases from the headspace at a consistent rate. After replacing the volatile gas with clean air, a second hollow needle equipped with a charcoal filter was added. The sampling interval was set at 2 s, and a flush time of 120 s was implemented to clear the system by replacing the air until the sensor signals returned to the baseline level. Additionally, a time-zero adjustment of 5 s was performed. The measurement time, pre-sampling time, and injection flow rate were 60 s, 8 s, and 400 mL/min, respectively.

### 2.9. Volatile Compounds

The volatile compounds were measured using the headspace solid-phase microextraction gas chromatography mass spectrometry (HS-SPME-GC-MS) described by Chen et al. [24], with slight modifications. Volatiles were extracted from a 2 g shiitake mushroom sample in a 20 mL glass vial. The vial was sealed promptly using a 20 mm aluminum pressure release seal, which was equipped with a PTFE/Silicone liner. After sealing, the vials were thermally equilibrated at 50 °C for 40 min. This specific equilibration time was chosen based on comparisons of the relative standard deviation, which indicated that the analysis achieved highly repeatable results at 50 min of equilibrium. A solid-phase microextraction fiber with a diameter of 65 μm and coated with divinylbenzene/polydimethylsiloxane was inserted into the vials through the PTFE/Silicone liner to conduct headspace analyses of volatile components present in the shiitake mushroom samples. The SPME fiber was then positioned within the headspace of the sample, with an exposure depth of 30 mm, slightly above the sample’s upper level. The SPME fiber was exposed to the headspace of the vials at room temperature (24 ± 1 °C) for 30 min. Extraction durations of 30 min yielded the optimal peak area response for the majority of volatile components. After performing headspace extraction, the SPME fibers were injected into the gas chromatograph and desorbed volatile compounds for 10 min under the splitless mode at a temperature of 250 °C.

Volatile compounds were analyzed using an Agilent 7890B GC coupled with an Agilent 5977B MS. The GC system was equipped with an HP-5MS fused silica capillary column (30 m × 0.25 mm I.D., 0.25 mm film thickness, Agilent Technologies, Santa Clara, CA, USA). The GC conditions were set as follows: helium was used as the carrier gas with a fixed flow rate of 1 mL/min. The injector temperature was set at 250 °C. The initial oven temperature programming was set at 35 °C for 3 min, then ramped at 3 °C/min to 150 °C, maintained for 1 min, and finally ramped at 5 °C/min to 220 °C, and held for 2 min. The ionization source temperature was set at 230 °C. The mass spectrometry (MS) analysis was performed using the electron impact mode at 70 eV, covering a range of 30 amu to 395 amu. Volatile compounds were identified with a database (NIST17.L).

### 2.10. Statistical Analysis

Each test group was conducted in triplicate, and the mean value was obtained. Three samples were randomly selected from each test group for analysis of physical properties and volatile odor characteristics. The experimental data from the drying process were analyzed using Excel 2013. The significance of the mean difference was evaluated using one-way analysis of variance (ANOVA) with Duncan’s new multiple range test. The significance level was set at 0.05, and the analysis was performed using SPSS 22.0 software.

## 3. Results and Discussion

### 3.1. Moisture Ratio

**Figure 1:**
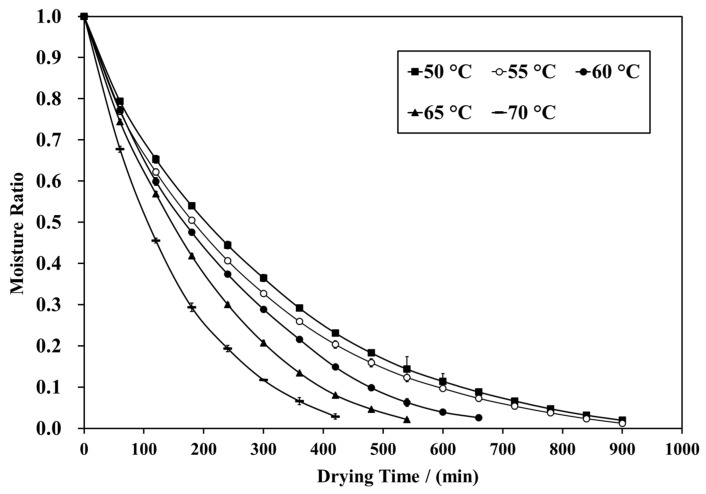
Curve of the moisture ratio of shiitake mushroom pilei.

To explore the effects of the FID temperature (50, 55, 60, 65, and 70 °C) on the drying kinetics of shiitake mushrooms, the moisture ratio (MR) curves versus the drying time of shiitake mushrooms at different FID temperatures are presented in Figure 1. The moisture content of the shiitake mushrooms was significantly influenced by the FID temperature. In Figure 1, the drying times for reducing the moisture content of shiitake mushrooms from the initial value of 8.73 kg/kg on a dry basis to the final value of 0.17 kg/kg on a dry basis were 900, 900, 660, 540, and 420 min at FID temperatures of 50, 55, 60, 65, and 70 °C, respectively. As the drying temperature increases, the drying rate accelerates, leading to a reduction in the drying time. Similar results have been reported by Stephenus et al. for *Phaleria macrocarpa* (Mahkota Dewa) fruits [18] and by Xie et al. for wolfberry [12]. The penetrating heat of FID prevents the surface hardening of the material, consequently enhancing the rate of moisture migration.

### 3.2. Shrinkage Ratio and Rehydration Ratio

**Figure 2:**
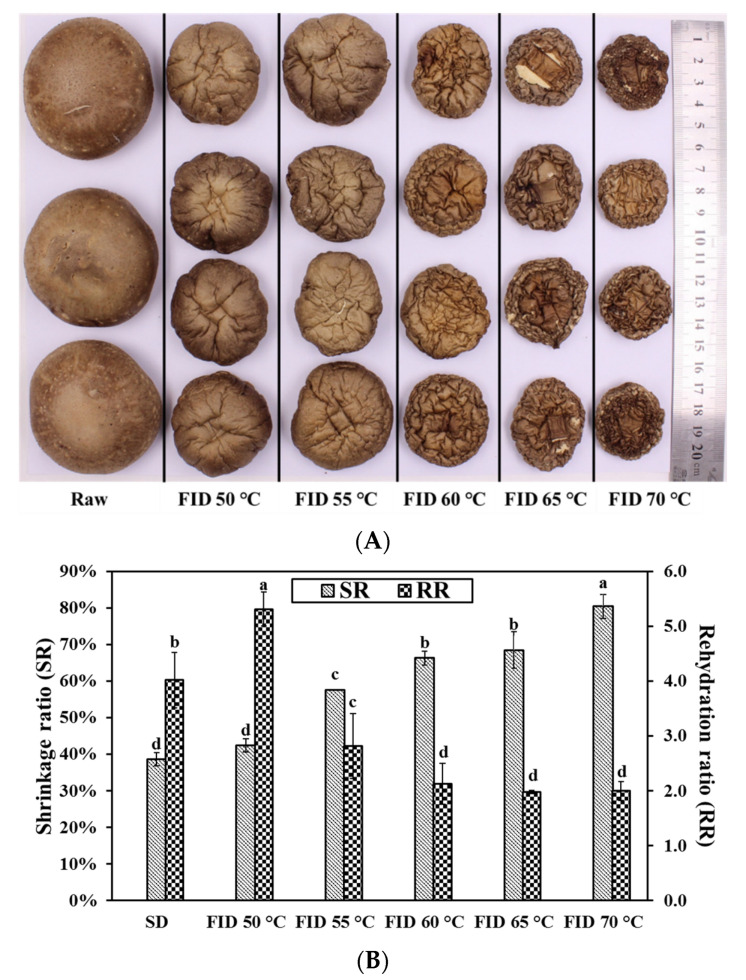
Real state changes of shiitake mushroom pilei under FID (**A**). Shrinkage and rehydration ratio of shiitake mushroom pilei under various drying conditions (**B**). Different letters represent significant differences (*p* < 0.05) between sample means.

During the process of drying, food materials experience alterations in volume, which can lead to either shrinkage or expansion, accompanied by the loss of water. The actual shrinkage images, shrinkage ratio (SR), and rehydration ratio (RR) of shiitake mushrooms under various drying conditions are presented in Figure 2. Shrinkage is a significant indicator that reflects the extent of damage to cell structures in vegetables, which may impact customer preferences [25]. The RR is a significant characteristic of dried shiitake mushrooms since they are typically consumed following rehydration [15]. The RR, which is the ratio of the weight of dried samples after rehydration to the weight of dried samples before rehydration, serves as a representative index for evaluating the microstructural quality during the drying process [3].

SD samples exhibit lower SR compared to FID. The phenomenon can be attributed to the longer drying time, allowing the product more time to undergo shrinkage [26]. As the drying temperature increased, the SR of shiitake mushrooms showed a significant increase, suggesting that high temperatures exacerbate the shrinkage of dried samples. This finding demonstrates that higher drying temperatures result in accelerated water removal, leading to increased stress in the matrix of the food material and the collapse of microscopic structures [27]. Similar phenomena have been observed in jujube slices dried using hot air drying, in which high temperatures lead to significant volume shrinkage [28]. Singh et al. also reported that increasing the drying temperature resulted in an increased shrinkage of Elephant Foot Yam at the same final moisture content [29].

Generally, SD samples exhibit a higher RR than FID counterparts except FID 50 °C. This phenomenon can potentially be attributed to the moisture migration process during FID. From Figure 2B, it is revealed that the drying temperature has a considerable impact on the RR of dried shiitake mushrooms. The RR decreased from 5.31 to 2.00 when the drying temperature was increased from 50 to 70 °C. The rehydration capacity decreases with the increased drying temperature during FID, which can be attributed to structural damage and cell shrinkage during the drying process [30]. At an appropriate drying temperature, moisture is gently and consistently removed from the inside to the surface, preserving the integrity of the moisture diffusion pathways and the structure. High drying temperatures can result in the collapse of the internal structure, which can impede water penetration during the rehydration process since the macroscopic properties of the product are determined by its microstructure. This finding is consistent with the results reported by Xie et al., who observed a significantly greater rehydration ability in wolfberry dried at 60 °C compared to those dried at 70 °C [12].

### 3.3. Microstructure

**Figure 3:**
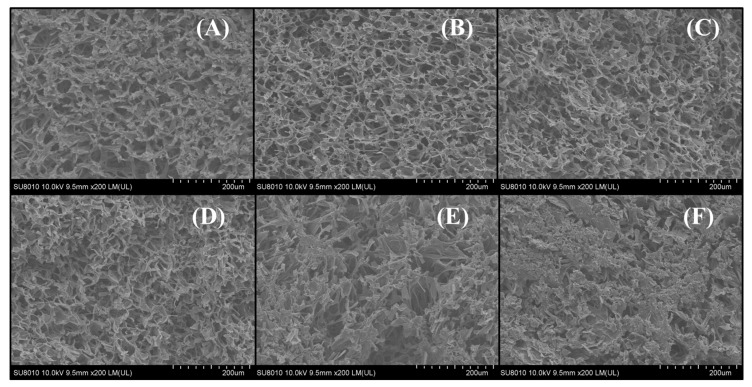
Surface microstructure of shiitake mushroom pilei under SD (**A**) and under FID at 50, 55, 60, 65, and 70 °C, respectively (**B**–**F**).

Microstructural characterization plays a vital role in evaluating the quality of final products. The microstructure of shiitake mushrooms’ longitudinal sections dried at various temperatures during FID were characterized using scanning electron microscopy (SEM) at a magnification level of 200-fold in Figure 3. As shown in Figure 3, the hyphae tissue is interlaced mutually towards various angles and directions in the absence of rigid cell walls for support [31]. All dried samples have abundant pores and a honeycomb-like microstructure. From Figure 3A, it can be found that the SD sample consisted of regular and loose cellular tissue structures. From Figure 3B–F, the shiitake mushroom hyphae exhibited varying degrees of shrinkage and collapse with the increasing drying temperature. The surface of the FID 50 °C sample (Figure 3B) exhibits a dense and complete porous tissue structure. The samples subjected to FID showed different levels of tissue structural collapse on their surfaces (Figure 3B–F). With the increase in drying temperature, visible contraction and tightness was observed in the hyphae. The collapse of the hyphae’s structure leads to the expansion of the free space between the hyphae. The collapse area increased with a higher drying temperature, and the largest collapse area was observed at 70 °C. This phenomenon can be explained via a significantly higher rate of surface water evaporation in contrast to moisture transfer from the interior to the surface. This result is in line with the results exhibited by RR, which indicate that higher structural integrity results in better rehydration capacity. Generally, the macroscopic properties of the biomaterial, including drying characteristics and rehydration capacity, are influenced by its microstructure [32].

### 3.4. Surface Color

**Figure 4:**
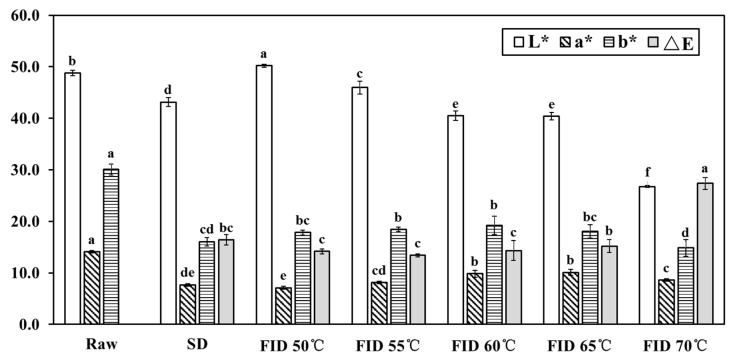
Colorimetric parameters of raw and dried shiitake mushroom pilei. Lowercase letters above the columns indicate the significant differences (*p* < 0.05) between sample means.

The surface color of shiitake mushrooms is a crucial quality indicator that determines consumer acceptance and market value [33]. The color parameters L*, a*, b*, and Δ*E* of samples under different FID temperatures are displayed in Figure 4. Generally, the dried sample showed lower lightness (L* value), redness (a* value), yellowness (b* value), and a higher total color difference (Δ*E* value) compared to the raw sample, which is consistent with previous studies [26]. Moreover, significant differences (*p* < 0.05) were observed in the L*, a*, b*, and Δ*E* values among the different FID temperatures. Higher drying temperatures employed in the FID process were found to be associated with a decreasing L* value and an increasing Δ*E* value. This result can be primarily attributed to the occurrence of the nonenzymatic Maillard reaction, known for promoting the formation of brown pigments. The Maillard reaction is influenced by multiple factors, such as pH, moisture content, temperature, and the ratio of amino acids to reducing sugars [34]. This was similar to the previous study conducted by Yang et al. [9], who found that an increase in browning resulted from higher temperature thermal processes.

### 3.5. Volatile Odor Profile Distinction

The electronic nose rapidly captures a wide range of volatile components and sensory attributes, simulating the human olfactory system through a sensor array system [35]. It has been implemented to classify and monitor the drying process of shiitake mushrooms [17]. Principal component analysis (PCA) is a multivariate chemometric method utilized for discerning correlation patterns among constituent variables that contribute to the differentiation of samples. Additionally, PCA can enhance visualization and highlight distinctions in volatile odor profiles [36].

**Figure 5:**
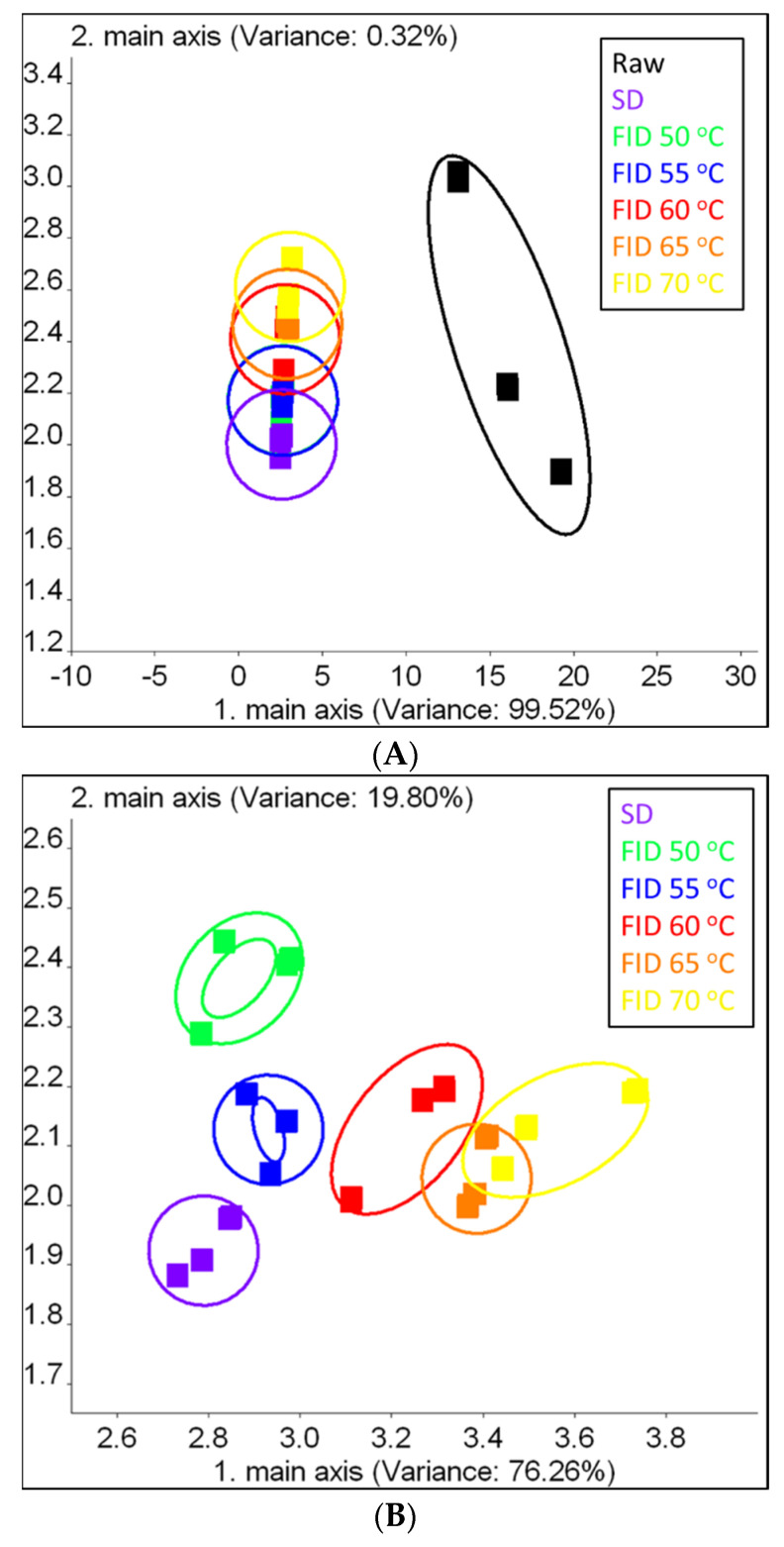
PCA (**A**,**B**) of electronic nose data from shiitake mushrooms.

The relationship between samples and electronic nose sensors was further elucidated through the application of PCA analysis, with the results presented in Figure 5A,B. The graph in Figure 5A illustrates that the first two principal components accounted for an accumulation variance contribution rate of 99.84% (with PC1 contributing 99.52% and PC2 contributing 0.32%). This result confirms the feasibility of PCA and establishes these two primary components as containing the majority of information relating to volatile compounds [37]. From Figure 5A, the obvious trend of separation between raw samples and dried samples with different drying conditions can be seen. The scores of raw samples are displayed on the right side, while the scores of dried samples are presented on the left side. The volatile odor profiles in the SD and FID samples differed significantly from those found in the raw samples. Additionally, the results indicated that the drying processes had an impact on the volatile odor profiles of shiitake mushrooms [24].

To further distinguish the profiles of the samples subjected to FID and SD, the electronic nose information of raw samples was excluded, and PCA analysis was performed, as depicted in Figure 5B. Despite the presence of sensor instability among replications, the volatile profiles obtained from different samples were classified into six distinct groups using the PCA algorithm, which indicated that the volatile odors of samples under different drying conditions can be distinguished. The first two principal components, PC1 and PC2, contributed 76.26% and 19.80% to the variance contribution rates, respectively. The cumulative contribution rate of 96.06% indicates that the first two principal components have captured the majority of information from the samples.

### 3.6. Volatile Compounds

**Figure 6:**
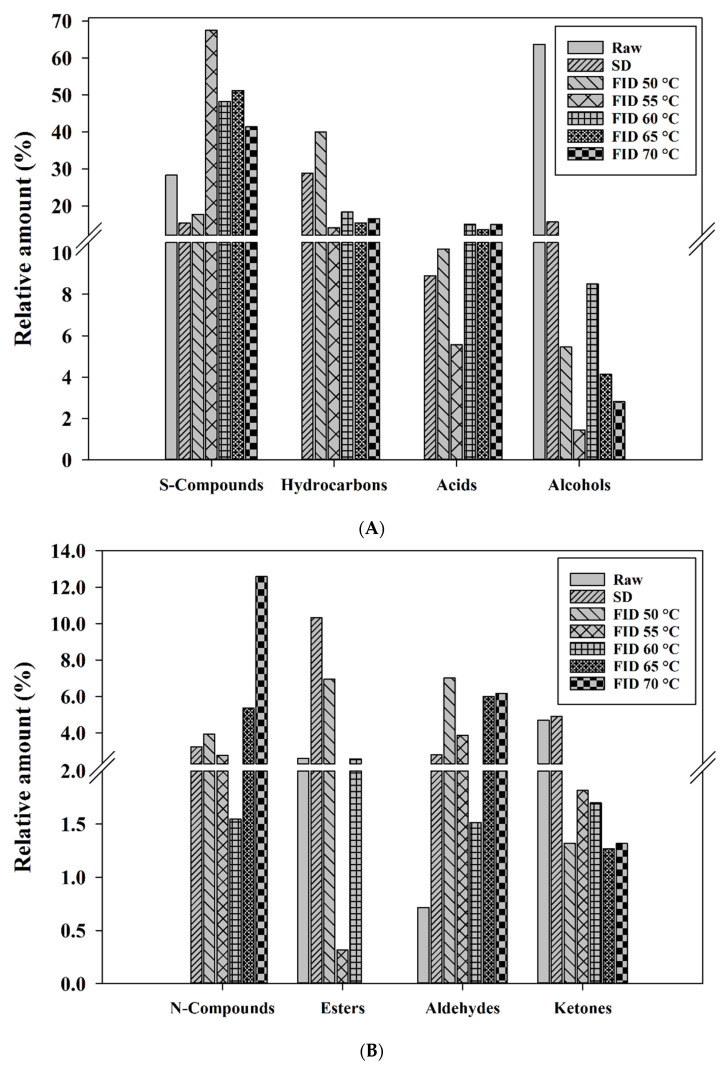
Relative amount of S-compounds, hydrocarbons, acids, alcohols (**A**), and N-compounds, esters, aldehydes, ketones (**B**) in raw and different dried shiitake mushrooms.

The characteristic aromas of food are primarily the result of volatile compounds, which are a prominent characteristic of shiitake mushrooms and have a significant impact on consumer preferences [38]. To investigate the impact of FID on the flavor profile of shiitake mushrooms, HS-SPME-GC-MS was utilized to analyze the distinct volatile compounds found in samples. As shown in Figure 6, these volatile components are classified as sulfur compounds (S-compounds), hydrocarbons, acids, alcohols, nitrogen compounds (N-compounds), esters, aldehydes, ketones, and others. The samples were found to contain a variety of volatile compounds, as summarized in Table 1. It is worth noting that the different types of samples exhibited variations in the types and concentrations of volatile components. A total of 103 volatile components were analyzed in shiitake mushrooms. These components included 10 S-compounds, 20 hydrocarbons, 5 acids, 7 alcohols, 20 N-compounds, 12 esters, 9 aldehydes, 7 ketones, and 13 other compounds. Among these, 12 different kinds of volatile compounds were examined in raw shiitake mushrooms, 41 compounds in SD, 46 compounds in FID 50 °C, 37 compounds in FID 55 °C, 40 compounds in FID 60 °C, 33 compounds in FID 65 °C, and 43 compounds in FID 70 °C. The FID treatment increased in the number of volatile compounds [26], while the overall content of volatile compounds decreased.

The alcohols (63.69%), S-compounds (28.28%), and ketones (4.70%) were the main volatile flavor components in raw shiitake mushrooms, which was consistent with previous studies [17]. The main volatile flavor components in dried shiitake mushrooms were S-compounds (15.33~67.47%), acids (5.56~14.96%), N-compounds (2.77~12.59%), aldehydes (2.79~7.02%), and esters (0.00~10.32%). Previous studies have reported that alcohols, aldehydes, and ketones are the predominant volatile compounds found in raw shiitake mushrooms, and they are known to significantly contribute to the flavor profiles of various foods [39,40,41]. The levels of alcohols, ketones, and hydrocarbons experienced a significant reduction following the drying process shown in Figure 6. A similar finding was recorded in other mushrooms [42,43]. Alcohols are the most abundant volatile components in raw shiitake mushrooms, and their levels obviously decrease after various drying treatments, especially at FID 55 °C and FID 70 °C. This indicates a close relationship between alcohol content and drying time and temperature. Thus, the samples exhibited higher concentrations of ketones and alcohols when subjected to a lower drying temperature. 1-octen-3-ol and 1-octen-3-one are important volatile eight-carbon compounds in shiitake mushrooms, presenting the flavor of mushrooms (Table 1). With the increase in FID temperature, a noticeable reduction in the concentration of these eight-carbon compounds is observed, which indicates that volatile eight-carbon compounds are thermosensitive components [26]. Meanwhile, these compounds are susceptible to enzymatic and non-enzymatic reactions, which may result in their degradation during the drying process [44].

The content of S-compounds, acids, N-compounds, and aldehyde exhibited varying degrees of increase after the FID treatment shown in Figure 6. The characteristic flavor of dried shiitake mushrooms is contributed by sulfur compounds. All samples were found to contain nine S-compounds, namely lenthionine, carbon disulfide, dimethyl sulfone, dimethyl trisulfide, 1,2,4,5-tetrathiane, 1,2,4-trithiolane, and 1,2,4,6-tetrathiepane, as identified through GC-MS analysis. It has been reported that straight-chain and cyclic S-compounds contribute significantly to the distinctive aroma of shiitake mushrooms [45]. Dimethyl trisulfide, a straight-chain sulfur compound, was detected in the dried samples. The main cyclic sulfur compounds detected in the dried samples were 1,2,4-trithiolane and lenthionine. Drying treatment can enhance the formation of straight-chain and cyclic sulfur compounds in shiitake mushrooms. Additionally, lenthionine undergoes degradation to produce dimethyl trisulfide [46], which involves enzymic reactions. Therefore, the variation in sulfur compounds among raw, SD, and FID shiitake mushrooms could be attributed to the enzymatic activities and drying temperatures involved in the drying processes. Organic acids contribute to the generation of sour and astringent tastes and play a crucial role in the flavor profile of shiitake mushrooms. Acetic acid is one of the main components of organic acids in shiitake mushrooms. The FID obviously enhances the formation of acetic acid in shiitake mushrooms (Table 1). This could be due to the degradation of proteins or amino acids [26], or it could be attributed to the increased enzyme activity resulting from the elevated temperature, leading to an increase in substrate yield [47]. The concentration of benzaldehyde, 2-methyl- butanal, and 3-methyl- butanal in the dried samples showed a significant increase compared to the raw samples (Table 1). This could be attributed to the oxidation and degradation of unsaturated fatty acids, which are the primary sources of aldehyde formation [48]. Furthermore, the elevated temperature of FID facilitates the occurrence of Maillard reactions [49], which increases the aldehyde content.

## 4. Conclusions

This study investigated the effects of far infrared radiation drying on the drying kinetics and quality characteristics (color, shrinkage ratio, rehydration ratio, microstructure, volatile odor profile distinction, and volatile compounds) of shiitake mushrooms. The drying time decreases with the increase in drying temperature in FID, and it has a smaller effect in the lower temperature range. The drying time decreases with increasing FID temperature, and the impact is less significant at lower temperatures. Increasing the drying temperature results in the shrinkage and collapse of the microstructure, reducing the rehydration rate, and emphasizing the impact of microstructure characteristics on macroscopic properties. The application of higher drying temperatures in the FID process is linked to a decrease in the L* value and an increase in the Δ*E* value. A PCA analysis can effectively distinguish the significant impact of FID on the volatile odor profiles of shiitake mushrooms. FID treatment enriched the samples with a greater variety of volatile compounds compared to raw shiitake mushrooms. Following FID processing, there was an increase in volatile components such as S-compounds, acids, N-compounds, and aldehydes, while volatile components like alcohols, ketones, and hydrocarbons decreased.

This study evaluated the effects of far infrared radiation drying on the physical properties and volatile odor characteristics of shiitake mushrooms. In future research, volatile and non-volatile flavor compounds should be studied to reveal their contributions to flavor during drying. Additionally, further exploration is needed to understand the impact of water migration processes, water status, and changes in cellular structure on flavor formation.

## Figures and Tables

**Table 1:** Contents of volatile compounds in raw and different dried shiitake mushrooms.

No.	Compounds	Formula	Areas (%)							Odorant Description
			Raw	SD	FID 50 °C	FID 55 °C	FID 60 °C	FID 65 °C	FID 70 °C	
	Alcohols		63.69%	15.62%	5.45%	1.44%	8.50%	4.14%	2.82%	
1	1-Butanol, 3-methyl-	C5H12O	nd	nd	0.88%	nd	nd	nd	nd	Malty, bitter
2	1-Decanol	C10H22O	nd	0.19%	nd	nd	0.25%	nd	nd	*
3	1-Dodecanol	C12H26O	nd	nd	nd	0.27%	nd	nd	nd	*
4	1-Octen-3-ol	C8H16O	62.33%	9.75%	2.21%	nd	0.82%	1.04%	1.42%	Mushroom, grass ^a^
5	1-Undecanol	C11H24O	nd	2.53%	nd	nd	0.42%	0.58%	0.54%	*
6	2-Octen-1-ol, (Z)-	C8H16O	1.36%	nd	nd	nd	nd	nd	nd	*
7	Phenylethyl Alcohol	C8H10O	nd	3.16%	2.36%	1.17%	7.01%	2.52%	0.86%	Sweet ^a^
	Ketones		4.70%	4.91%	1.32%	1.82%	1.70%	1.27%	1.32%	
8	1-Octen-3-one	C8H14O	4.08%	nd	nd	0.50%	nd	nd	nd	Mushroom
9	2,3-Pentanedione	C5H8O2	nd	nd	nd	nd	nd	nd	0.28%	Almond, butter, pungent ^a^
10	2-Undecanone	C11H22O	nd	1.50%	1.32%	nd	nd	0.96%	1.04%	Dusty, ketone, tallow ^a^
11	3-Octanone	C8H16O	0.62%	3.11%	nd	1.32%	0.65%	nd	nd	Earthy, mushroom ^a^
12	3-Octen-2-one	C8H14O	nd	nd	nd	nd	nd	0.31%	nd	*
13	Acetophenone	C8H8O	nd	0.31%	nd	nd	nd	nd	nd	Glue, musty ^a^
14	Furaneol	C6H8O3	nd	nd	nd	nd	1.04%	nd	nd	*
	Hydrocarbons		0.00%	28.81%	38.80%	14.10%	16.44%	15.38%	16.53%	
15	1-Tetradecene	C14H28	nd	nd	nd	0.76%	0.71%	nd	nd	*
16	Bicyclo [4.1.0]heptane, 3,7,7-trimethyl-, (1.alpha., 3.alpha., 6.alpha.)	C10H18	nd	nd	1.50%	nd	nd	nd	nd	*
17	Butane	C4H10	nd	nd	0.98%	nd	nd	nd	0.37%	*
18	Cetene	C16H32	nd	nd	1.26%	nd	nd	nd	nd	*
19	Cyclohexene,3-(1-methylpropyl)-	C10H18	nd	1.85%	3.49%	0.37%	nd	nd	0.28%	*
20	Cyclopentane, 1-methyl-1-(2-methyl-2-propenyl)-	C10H18	nd	0.90%	nd	nd	nd	nd	nd	*
21	Cyclopentane, nonyl-	C14H28	nd	nd	1.05%	nd	nd	nd	nd	*
22	Cyclopropane, propyl-	C6H12	nd	0.57%	nd	nd	nd	nd	nd	*
23	Cyclotetradecane	C14H28	nd	1.12%	nd	nd	nd	nd	nd	*
24	Cyclotridecane	C13H26	nd	nd	1.99%	nd	nd	nd	nd	*
25	Dodecane	C12H26	nd	8.07%	8.46%	4.32%	5.53%	5.44%	4.85%	Alkane ^a^
26	Heptylcyclohexane	C13H26	nd	0.59%	0.58%	nd	0.22%	nd	0.28%	*
27	n-Nonylcyclohexane	C15H30	nd	0.63%	0.74%	nd	0.22%	0.29%	0.42%	*
28	Nonane, 5-(2-methylpropyl)-	C13H28	nd	0.99%	nd	nd	nd	nd	nd	Linseed oil, oily, sweaty ^a^
29	Oxirane, trimethyl-	C5H10O	nd	0.55%	nd	nd	nd	nd	nd	*
30	Styrene	C8H8	nd	nd	2.18%	0.99%	0.87%	0.74%	1.36%	*
31	Tetradecane	C14H30	nd	nd	1.93%	0.78%	0.91%	0.81%	0.95%	*
32	Tetradecane, 3-methyl-	C15H32	nd	0.37%	nd	nd	nd	nd	nd	*
33	Tridecane	C13H28	nd	12.04%	13.61%	6.19%	7.05%	7.28%	7.38%	*
34	Undecane	C11H24	nd	1.13%	1.05%	0.68%	0.93%	0.82%	0.65%	*
	S-compounds		28.28%	15.33%	17.72%	67.47%	48.21%	51.18%	41.42%	
35	1,2,4,5-Tetrathiane	C2H4S4	8.53%	3.03%	3.27%	7.79%	4.45%	2.87%	nd	*
36	1,2,4,6-Tetrathiepane	C3H6S4	nd	0.86%	1.12%	2.21%	2.98%	2.27%	2.27%	*
37	1,2,4-Trithiolane	C2H4S3	3.37%	8.70%	9.43%	14.66%	17.06%	25.68%	24.62%	Garlic ^a^
38	2,4,5-trithiahexane 2,2-dioxide	C3H8O2S3	0.59%	nd	nd	nd	nd	nd	nd	*
39	Butane, 1-isothiocyanato-3-methyl-	C6H11NS	nd	nd	nd	1.58%	1.76%	1.84%	nd	*
40	Carbon disulfide	CS2	6.57%	1.46%	1.85%	11.68%	7.04%	8.49%	4.52%	*
41	Dimethyl sulfone	C2H6O2S	nd	nd	nd	nd	1.28%	3.23%	6.55%	*
42	Dimethyl trisulfide	C2H6S3	nd	nd	nd	8.99%	2.46%	nd	1.46%	Alliaceous, meaty ^a^
43	Lenthionine	C2H4S5	9.22%	1.28%	2.04%	16.53%	10.18%	6.81%	2.00%	Mushroom ^a^
44	Tetrasulfide, dimethyl	C2H6S4	nd	nd	nd	4.03%	0.99%	nd	nd	*
	Acids		0.00%	8.90%	10.20%	5.56%	15.01%	13.55%	14.96%	
45	Acetic acid	C2H4O2	nd	8.40%	8.81%	5.17%	15.01%	12.94%	14.12%	Vinegar-like ^a^
46	Butanoic acid	C4H8O2	nd	0.50%	nd	nd	nd	nd	nd	*
47	Butanoic acid, 2-methyl-	C5H10O2	nd	nd	0.21%	0.39%	nd	nd	nd	*
48	Butanoic acid, 3-methyl-	C5H10O2	nd	nd	0.24%	nd	nd	0.61%	0.84%	*
49	Pentanoic acid	C5H10O2	nd	nd	0.93%	nd	nd	nd	nd	*
	Aldehydes		0.72%	2.79%	7.02%	3.86%	1.51%	6.01%	6.17%	
50	2-Furancarboxaldehyde, 5-methyl-	C6H6O2	nd	nd	nd	nd	nd	nd	0.51%	*
51	2-Isopropyl-5-methylhex-2-enal	C10H18O	nd	nd	nd	nd	nd	0.46%	0.33%	*
52	2-Phenylpropenal	C9H8O	0.72%	nd	nd	nd	nd	nd	nd	*
53	Benzaldehyde	C7H6O	nd	2.79%	2.97%	2.80%	0.76%	1.52%	1.55%	Almond, bitter almond ^a^
54	Benzeneacetaldehyde, .alpha.-ethylidene-	C10H10O	nd	nd	nd	nd	nd	0.57%	0.42%	*
55	Butanal, 2-methyl-	C5H10O	nd	nd	0.90%	nd	0.20%	1.33%	1.24%	*
56	Butanal, 3-methyl-	C5H10O	nd	nd	1.26%	0.86%	0.56%	1.59%	1.34%	*
57	Hexanal	C6H12O	nd	nd	0.92%	0.21%	nd	0.53%	0.78%	Aldehyde, leaves, vinous ^a^
58	Propanal, 2-methyl-	C4H8O	nd	nd	0.97%	nd	nd	nd	nd	*
	N-compounds		0.00%	3.23%	3.93%	2.77%	3.37%	6.00%	12.59%	
59	1-Butanamine, 3-methyl-N-(3-methylbutylidene)-	C10H21N	nd	nd	nd	0.64%	nd	nd	nd	*
60	1-Butanamine, N-(2-furanylmethylene)-3-methyl-	C10H15NO	nd	nd	nd	0.97%	1.82%	0.98%	0.63%	*
61	1H-Isoindole, 3-methoxy-4,7-dimethyl-	C11H13NO	nd	nd	2.21%	nd	nd	nd	nd	*
62	1H-Pyrrole-2-carboxaldehyde, 1-ethyl-	C7H9NO	nd	0.31%	nd	nd	nd	nd	nd	*
63	2-(3-Methylbutyl)-3,5-dimethylpyrazine	C11H18N2	nd	nd	nd	nd	nd	nd	0.40%	*
64	2-Isoamyl-6-methylpyrazine	C10H16N2	nd	nd	nd	nd	nd	nd	0.30%	*
65	3-Acetyl-1H-pyrroline	C6H7NO	nd	0.33%	nd	nd	nd	nd	nd	*
66	4H-Pyran-4-one, 2,3-dihydro-3,5-dihydroxy-6-methyl-	C6H8O4	nd	nd	nd	nd	nd	0.64%	1.44%	*
67	Aziridine, 1-ethenyl-	C4H7N	nd	0.62%	nd	nd	nd	nd	nd	*
68	Ethanone, 1-(1H-pyrrol-2-yl)-	C6H7NO	nd	nd	nd	0.39%	0.68%	nd	nd	*
69	Formamide, N,N-dimethyl-	C3H7NO	nd	0.24%	0.64%	nd	0.26%	nd	nd	*
70	Pyrazine, 2,3-dimethyl-	C6H8N2	nd	nd	nd	nd	nd	nd	0.38%	*
71	Pyrazine, 2,5-dimethyl-	C6H8N2	nd	nd	nd	nd	nd	nd	1.74%	*
72	Pyrazine, 2,6-diethyl-	C8H12N2	nd	nd	nd	nd	nd	1.90%	nd	*
73	Pyrazine, 2-ethyl-6-methyl-	C7H10N2	nd	0.80%	0.89%	0.29%	0.60%	1.87%	2.22%	*
74	Pyrazine, 3,5-diethyl-2-methyl-	C9H14N2	nd	nd	nd	nd	nd	nd	0.40%	*
75	Pyrazine, 3-ethyl-2,5-dimethyl-	C8H12N2	nd	nd	nd	0.48%	nd	nd	3.48%	*
76	Pyrazine, methyl-	C5H6N2	nd	0.26%	0.19%	nd	nd	nd	0.34%	*
77	Pyrazine, trimethyl-	C7H10N2	nd	nd	nd	nd	nd	0.61%	1.27%	*
78	Triethylamine	C6H15N	nd	0.67%	nd	nd	nd	nd	nd	*
	Esters		2.61%	10.32%	6.95%	0.32%	2.24%	0.00%	0.00%	*
79	1,2-Benzenedicarboxylic acid, bis(2-methylpropyl) ester	C16H22O4	nd	nd	0.47%	nd	nd	nd	nd	*
80	2(3H)-Furanone, dihydro-5-methyl-	C5H8O2	nd	2.09%	5.66%	nd	0.78%	nd	nd	*
81	2(3H)-Furanone, dihydro-5-pentyl-	C9H16O2	nd	0.72%	nd	nd	nd	nd	nd	*
82	2,2,4-Trimethyl-1,3-pentanediol diisobutyrate	C16H30O4	nd	nd	nd	0.32%	0.40%	nd	nd	*
83	Butyrolactone	C4H6O2	nd	6.70%	nd	nd	nd	nd	nd	*
84	Cyclohexene,3-(1-methylpropyl)-	C10H18	nd	nd	nd	nd	0.35%	nd	nd	*
85	Dibutyl phthalate	C16H22O4	nd	0.45%	nd	nd	nd	nd	nd	*
86	Dimethyl phthalate	C10H10O4	nd	nd	0.50%	nd	0.39%	nd	nd	*
87	Formic acid, octyl ester	C9H18O2	2.61%	nd	nd	nd	nd	nd	nd	*
88	Phthalic acid, butyl hept-4-yl ester	C19H28O4	nd	nd	0.33%	nd	nd	nd	nd	*
89	Phthalic acid, hept-4-yl isobutyl ester	C19H28O4	nd	0.36%	nd	nd	nd	nd	nd	*
90	Propanoic acid, 2-methyl-, 2-phenylethyl ester	C12H16O2	nd	nd	nd	nd	0.32%	nd	nd	*
	Others		0.00%	10.08%	8.61%	2.65%	3.01%	2.47%	4.18%	
91	(3aS,8aS)-6,8a-Dimethyl-3-(propan-2-ylidene)-1,2,3,3a,4,5,8,8a-octahydroazulene	C15H24	nd	nd	1.14%	0.00%	0.33%	nd	nd	*
92	1H-Indene, 1-ethylidene-	C11H10	nd	nd	nd	0.29%	nd	nd	nd	*
93	Benzene, 1,2-dichloro-	C6H4Cl2	nd	nd	4.58%	1.32%	1.94%	1.38%	2.15%	*
94	Benzene, 1,4-dichloro-	C6H4Cl2	nd	8.42%	nd	nd	nd	nd	nd	*
95	Diisobutyl cellosolve	C10H22O2	nd	nd	nd	0.00%	nd	nd	nd	*
96	Dimethyl ether	C2H6O	nd	nd	0.76%	0.20%	nd	nd	nd	*
97	Ethylbenzene	C8H10	nd	nd	0.31%	nd	nd	nd	nd	*
98	Furan, 2-pentyl-	C9H14O	nd	nd	nd	0.85%	nd	1.09%	1.51%	*
99	Naphthalene, 1,4-dimethyl-	C12H12	nd	nd	0.70%	nd	nd	nd	nd	*
100	Naphthalene, 1,6-dimethyl-	C12H12	nd	nd	0.21%	nd	nd	nd	nd	*
101	Naphthalene, 2-methyl-	C11H10	nd	nd	0.91%	nd	0.36%	nd	nd	*
102	Phthalic acid, 4-fluoro-2-nitrophenyl methyl ester	C15H10FNO6	nd	nd	nd	0.00%	nd	nd	nd	*
103	p-Xylene	C8H10	nd	1.66%	nd	nd	0.38%	nd	0.52%	*

nd: Not detected for the compound. ^a^ the aroma descriptions originate from “the LRI and Odour Database” (http://www.odour.org.uk/index.html (accessed on 21 August 2023)). *: The aroma of this compound cannot be described in simple terms.

## Data Availability

Data is contained within the article.
